# Patient–ventilator asynchrony in acute brain-injured patients: a prospective observational study

**DOI:** 10.1186/s13613-020-00763-8

**Published:** 2020-10-19

**Authors:** Xu-Ying Luo, Xuan He, Yi-Min Zhou, Yu-Mei Wang, Jing-Ran Chen, Guang-Qiang Chen, Hong-Liang Li, Yan-Lin Yang, Linlin Zhang, Jian-Xin Zhou

**Affiliations:** Department of Critical Care Medicine, Beijing Tiantan Hospital, Capital Medical University, Fengtai District, No. 119, South 4th Ring West Road, Beijing, 100070 China

**Keywords:** Mechanical ventilation, Asynchrony, Prevalence, Severity, Monitoring, Brain injury

## Abstract

**Background:**

Patient–ventilator asynchrony is common in mechanically ventilated patients and may be related to adverse outcomes. Few studies have reported the occurrence of asynchrony in brain-injured patients. We aimed to investigate the prevalence, type and severity of patient–ventilator asynchrony in mechanically ventilated patients with brain injury.

**Methods:**

This prospective observational study enrolled acute brain-injured patients undergoing mechanical ventilation. Esophageal pressure monitoring was established after enrollment. Flow, airway pressure, and esophageal pressure–time waveforms were recorded for a 15-min interval, four times daily for 3 days, for visually detecting asynchrony by offline analysis. At the end of each dataset recording, the respiratory drive was determined by the airway occlusion maneuver. The asynchrony index was calculated to represent the severity. The relationship between the prevalence and the severity of asynchrony with ventilatory modes and settings, respiratory drive, and analgesia and sedation were determined. Association of severe patient–ventilator asynchrony, which was defined as an asynchrony index  ≥ 10%, with clinical outcomes was analyzed.

**Results:**

In 100 enrolled patients, a total of 1076 15-min waveform datasets covering 330,292 breaths were collected, in which 70,156 (38%) asynchronous breaths were detected. Asynchrony occurred in 96% of patients with the median (interquartile range) asynchrony index of 12.4% (4.3%–26.4%). The most prevalent type was ineffective triggering. No significant difference was found in either prevalence or asynchrony index among different classifications of brain injury (*p *> 0.05). The prevalence of asynchrony was significantly lower during pressure control/assist ventilation than during other ventilatory modes (*p *< 0.05). Compared to the datasets without asynchrony, the airway occlusion pressure was significantly lower in datasets with ineffective triggering (*p *< 0.001). The asynchrony index was significantly higher during the combined use of opioids and sedatives (*p *< 0.001). Significantly longer duration of ventilation and hospital length of stay after the inclusion were found in patients with severe ineffective triggering (*p *< 0.05).

**Conclusions:**

Patient–ventilator asynchrony is common in brain-injured patients. The most prevalent type is ineffective triggering and its severity is likely related to a long duration of ventilation and hospital stay. Prevalence and severity of asynchrony are associated with ventilatory modes, respiratory drive and analgesia/sedation strategy, suggesting treatment adjustment in this particular population.

*Trial registration* The study has been registered on 4 July 2017 in ClinicalTrials.gov (NCT03212482) (https://clinicaltrials.gov/ct2/show/NCT03212482).

## Background

Patient–ventilator asynchrony refers to a mismatch between the patient’s demand and ventilatory support, which can occur when the patient’s respiratory drive is either relatively high or low [1, 2]. Patient–ventilator asynchrony is common in mechanically ventilated patients and severe asynchrony may be related to adverse outcomes, including prolonged duration of mechanical ventilation, a higher rate of weaning failure, and even higher hospital mortality [3].

The majority of studies investigate the relationship between patient–ventilator asynchrony and ventilatory modes and settings [4]. Additionally, the prevalence and type of asynchrony may also be related to the patient’s characteristics [3]. For example, patients with obstructive diseases are likely with a high incidence of ineffective triggering [5], in part due to dynamic hyperinflation and intrinsic end-expiratory positive pressure (PEEP) [6]; while, in patients with acute respiratory distress syndrome with extremely low compliance, premature cycling is the most frequent type [7]. Respiratory drive and rhythm are controlled by neurons in the brainstem, which may also be influenced by cortical inputs [8]. Therefore, brain-injured patients may have had a high risk of patient–ventilator asynchrony. However, to date, no study focused on the prevalence and type of asynchrony, as well as the severity and related factors in brain-injured patients.

The prevalence of patient–ventilator asynchrony is also affected by the method of detection. The ventilator waveforms, including flow and airway pressure, are usually used to identify asynchrony, but the accuracy is relatively low in a certain type of asynchrony [9, 10]. As an alternative to pleural pressure, esophageal pressure monitoring, combined with flow and airway pressure waveforms analysis, can help to detect and classify asynchrony more accurately [11, 12].

In this prospective observational study, acute brain-injured patients undergoing invasive mechanical ventilation were included, and esophageal pressure monitoring was established to facilitate the diagnosis of asynchrony. Our primary purpose was to comprehensively investigate the type, prevalence, and severity of asynchrony in this population. Association of asynchrony with respiratory drive, ventilatory mode and setting, and brain injury classification were also determined.

## Methods

### Study setting and population

This prospective observational study was conducted in the intensive care unit (ICU) of Beijing Tiantan Hospital, Capital Medical University. We included mechanically ventilated patients with acute brain injury, which was defined a priori as traumatic brain injury, stroke (ischemic stroke, spontaneous intracerebral hemorrhage and subarachnoid hemorrhage) and post-craniotomy for brain tumor [13, 14]. Exclusion criteria were as follows: (1) age younger than 18 years old; (2) anticipated duration of mechanical ventilation less than 24 h; (3) presented with epilepsy; (4) presented with agitation; (5) contraindications for esophageal balloon catheter insertion which included evidence of severe coagulopathy, diagnosed or suspected esophageal varices, and history of esophageal, gastric or lung surgery; (6) evidence of active air leak from the lung which included bronchopleural fistula, pneumothorax, pneumomediastinum, and an existing chest tube; (7) moribund conditions with a low likelihood of survival for more than 24 h; and (8) refusal to participate.

The study protocol was approved by the institutional review boards of Beijing Tiantan Hospital, Capital Medical University (KY 2017-028-02), and was registered at ClinicalTrials.gov on July 4, 2017 (NCT03212482). Written informed consent was obtained from the patient or appropriate substitute decision-makers.

### Esophageal pressure monitoring

After the patient enrollment, an AVEA ventilator (CareFusion Co., USA) was applied. The nasogastric tube was replaced by an esophageal balloon catheter (SmartCath-G catheter: LOT 7003300, CareFusion Co., San Diego, CA, USA) which can provide esophageal pressure monitoring and tube feeding simultaneously.

Before the catheter insertion, a balloon leak test was performed using the Esophageal Maneuver function on the ventilator. In order to reflect the pleural pressure accurately, the esophageal balloon was placed in the lower two-thirds of the intrathoracic esophagus. The balloon volume was determined by the ventilator automatically. Then the optimal balloon position was adjusted by Baydur’s occlusion test in patients with spontaneous breathing [15] or positive pressure occlusion test in patients during passive ventilation [16].

### Ventilator mode selection and settings in routine clinical practice

The investigators did not involve in the treatment of the patients. The selection of ventilator modes and settings was decided by the responsible ICU physicians and remained unchanged during waveform data collection.

In our unit, assist/control modes, either pressure assist/control ventilation (PACV) or volume assist/control ventilation (VACV), are usually initiated in patients with acute hypoxemic respiratory failure. The ventilator mode is switched to pressure support ventilation (PSV) as long as the patient triggers all ventilator breaths during assist/control ventilation. If the backup ventilation is induced during PSV, pressure-preset synchronized intermittent mandatory ventilation plus pressure support (SIMV + PS) is used. The tidal volume is set as 6–8 ml/kg predicted body weight, which is obtained by adjusting inspiratory pressure during pressure-preset ventilation. The respiratory rate is usually set to maintain an arterial partial pressure of carbon dioxide of 35–40 mmHg as long as possible. The inspiratory trigger sensitivity is usually set as 1–2 L/min for flow-trigger and 1.5–3 cmH_2_O for pressure-trigger. During VACV, a decelerating flow with a peak flow between 45 and 60 L/min is set to obtain an inspiratory-to-expiratory ratio of 1:1.5–2. During PSV, a flow cycling is usually set as 25% of peak inspiratory flow. Inspired oxygen fraction and PEEP are set according to the patient’s oxygenation status.

### Data collection

Clinical data were collected at the study entry, including demographic data, Acute Physiology and Chronic Health Evaluation (APACHE) II score at the ICU admission, classification of brain injury, the ratio of the arterial partial pressure of oxygen to inspired oxygen fraction and the Sequential Organ Failure Assessment (SOFA) score at the inclusion, and duration of mechanical ventilation before the inclusion.

Flow–time, airway pressure–time and esophageal pressure–time waveforms were collected with a laptop via a dedicated acquisition system (VOXP Research Data Collector 3.2, Applied Biosignals GmbH, Weener, Germany) at 100 Hz for offline analysis. The signals were recorded four times daily (at 03:00 AM, 09:00 AM, 15:00 PM, and 21:00 PM), each for 15 min and saved as one waveform dataset. We collected ventilatory modes and settings, physiological variables, the use of sedatives, analgesics and neuromuscular blocking agents at the beginning of waveform recording. After the dataset collection, the Glasgow Coma Scale (GCS) and the Sedation-Agitation Scale (SAS) were evaluated. Arterial blood gas analysis was also collected at least once per day for each patient after the recording. The investigators were not involved in the treatment of the patients. Ventilatory modes and settings, as well as sedation and analgesia, were decided by the responsible ICU physicians and remained unchanged during the waveform data collection. At 30 min prior to waveform recording, the airway was suctioned. During the recording, unnecessary suctioning and physical therapy were avoided. The waveform dataset collection was conducted for 3 days as long as the patient was not weaned from the ventilator.

At the end of each waveform dataset recording, end-expiratory occlusion was performed. For patients with spontaneous breathing, the airway occlusion pressure (P_0.1_) was measured and averaged for five occlusions to represent the respiratory drive [17].

Patients were followed up until 60 days after the enrollment, hospital discharge or death, whichever occurred first. Duration of mechanical ventilation after the inclusion, length of stay in the ICU and hospital after the inclusion, the Glasgow Outcome Scale (GOS), and all-cause mortality were documented.

### Definition of patient–ventilator asynchrony

Seven asynchrony patterns were determined through a priori definition, including ineffective triggering, double-triggering, auto-triggering, flow insufficiency, premature cycling, delayed cycling, and reverse triggering [1, 2, 18–20]. The detailed description and example waveforms are shown in Additional file 1.

The collected waveform datasets were offline analyzed independently by the two investigators (XYL and XH). All breaths were inspected and labeled as no patient–ventilator asynchrony or one of the seven types of asynchrony defined above. The diagnosis of the patient–ventilator asynchrony was confirmed as the same decisions were made by the two investigators. When the two readings were discrepant, the final decision was made based on a group discussion (HLL, YLY and JXZ). Investigators taking part in the diagnosis of asynchrony were blinded to the patient’s clinical data and outcome data.

Overall and each type of asynchronous breath were counted. The prevalence of patient–ventilator asynchrony was calculated on the basis of all collected breaths and enrolled patients. The severity of the patient–ventilator asynchrony was represented by the asynchrony index, which is equal to the number of asynchronous breaths divided by the total number of breaths collected and multiplied by 100% [21]. The asynchrony index was calculated in each 15-min waveform dataset and in each patient after merging the collected datasets into a single recording session. Severe patient–ventilator asynchrony was defined as an asynchrony index  ≥ 10% in either dataset-based or patient-based analysis [22, 23].

### Statistical analysis

Inter-observer reliability for detecting asynchrony was evaluated using a weighted kappa statistic and 95% confidence interval (CI). The prevalence and 95% CI of patient–ventilator asynchrony were calculated in all collected breaths and patient-based analysis. The present study was a descriptive exploratory study; therefore, no formal sample size calculation was performed. We planned to enroll 100 patients with anticipating 1000 15-min waveform datasets collection.

Continuous variables were presented as the median and interquartile range (IQR) and categorical variables as numbers and percentages. The prevalence of asynchrony was compared among different classifications of brain injury by the Chi square test. The asynchrony index was compared among different ventilatory modes and different analgesia and sedation strategies by the Kruskal–Wallis test. P_0.1_ was also compared among different types of asynchrony by the Kruskal–Wallis test. All pairwise comparisons were performed with Bonferroni correction. The asynchrony index between dataset with SAS ≤ 2 and ≥ 3, as well as between datasets with GCS ≤ 8 and ≥ 9, was compared by Mann–Whitney U-test.

Associated factors with the severe ineffective triggering were analyzed by the Mann–Whitney U-test for continuous variables and the Chi square test or Fisher’s exact test for categorical variables.

All analysis was performed using the statistical software package SPSS 21 (SPSS Inc., Chicago, IL, USA). A *p* value of less than 0.05 was considered to be significant.

## Results

**Fig. 1:**
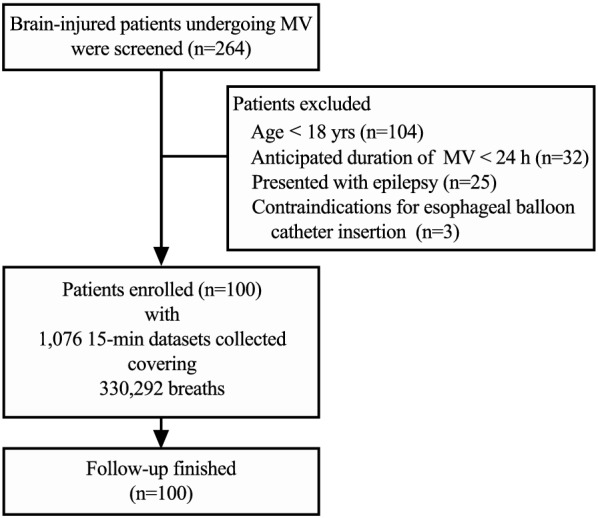
Patients flow-chart

From June 2017 to July 2019, 100 patients with a primary diagnosis with stroke (44%), post-craniotomy for brain tumors (37%), and traumatic brain injury (19%) were enrolled (Fig. 1). Table 1 shows the characteristics of the patients. The offline data analysis was completed in February 2020. The evaluation of inter-observer reliability for the detection of asynchrony revealed weighted kappa (95% CI) ranging from 0.930 (0.927–0.933) to 0.991 (0.987–0.994) for the seven types of patient–ventilator asynchrony (Additional file 1: Table S1).

**Table 1:** Characteristics of the patients

Characteristics	*N *= 100
Male sex *n* (%)	67 (67)
Age (years)	53 (39–64)
Body mass index (kg/m^2^)	24.2 (21.3–26.4)
Classification of brain injury, *n* (%)	
Stroke	44 (44)
Post-craniotomy for brain tumors	37 (37)
Traumatic brain injury	19 (19)
Acute hypoxemic respiratory failure, *n* (%)	76 (76)
History of chronic obstructive pulmonary diseases, *n* (%)	2 (2)
APACHE II at ICU admission	18 (14–21)
SOFA at inclusion	6 (4–7)
GCS at inclusion	10 (5–11)
PaO_2_/FiO_2_ ratio at inclusion (mmHg)	231 (176–308)
Duration of mechanical ventilation before inclusion (hours)	19 (15–22)
Duration of mechanical ventilation after inclusion (days)	7 (5–15)
Length of ICU stay after inclusion (days)	16 (10–27)
Length of hospital stay after inclusion (days)	31 (24–42)
GOS at the end of follow-up	3 (2–3)
All-cause mortality, *n* (%)	16 (16)

Categorical variables are shown as number (percentage); continuous variables are shown as median (interquartile range)

*APACHE* Acute Physiology and Chronic Health Evaluation, *FiO*_*2*_ inspired oxygen fraction, *GCS* Glasgow Coma Scale, *GOS* Glasgow Outcome Scale, *ICU* Intensive care unit, *PaO*_*2*_ Arterial partial pressure of oxygen, *SOFA* Sequential Organ Failure Assessment

### Prevalence, type and severity of patient–ventilator asynchrony

**Fig. 2:**
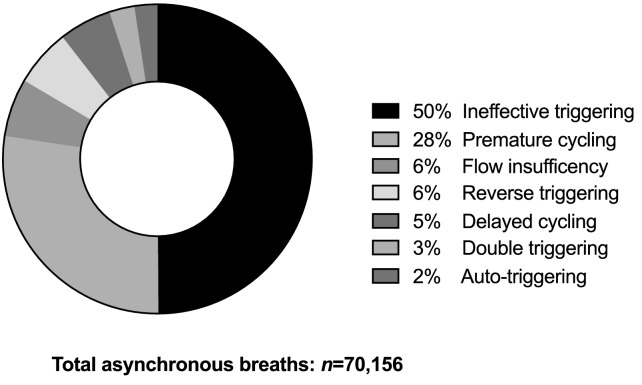
The proportion of different types of patient–ventilator asynchrony in breath-based analysis. A total of 70,156 asynchronous breaths were detected

Among the included patients, a total of 1076 15-min waveform datasets covering 330,292 breaths were collected and analyzed, in which 70,156 (38%, 95% CI 35%–40%) asynchronous breaths were detected. Ineffective triggering (50%) and premature cycling (28%) comprised the majority of asynchrony types (Fig. 2).

**Fig. 3:**
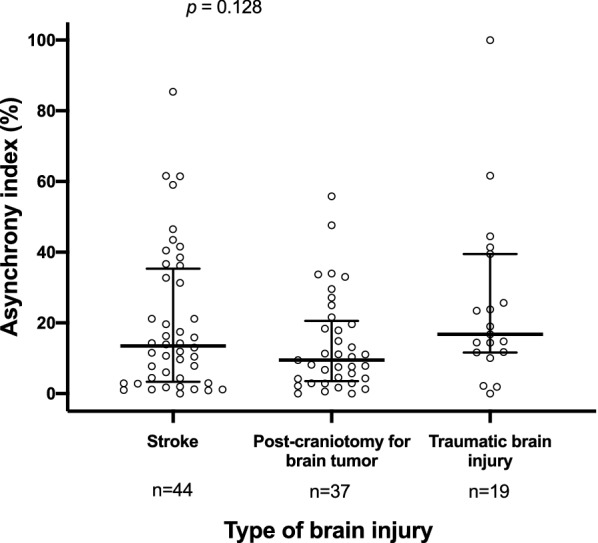
No significant difference was found in asynchrony index among the three classifications of brain injury (*p* = 0.128). Individual data, median, and interquartile ranges are shown. The median (interquartile range) asynchrony index was 13.5% (3.3%–35.3%), 9.5% (3.5%–20.6%), and 16.8% (11.7%–39.5%) in patients with stroke, post-craniotomy for brain tumor, and traumatic brain injury, respectively

After merging datasets into one single session in each patient, asynchrony occurred in 96 (96%, 95%CI 92%–100%) patients, with the most prevalent type of ineffective triggering followed by double-triggering, premature cycling, delayed cycling, and flow insufficiency (Table 2). The median (IQR) asynchrony index was 12.4% (4.3%–26.4%) (Additional file 1: Table S2). No significant difference was found in either prevalence or asynchrony index among the three classifications of brain injury (Table 2 and Fig. 3).

**Table 2:** Prevalence of asynchrony in enrolled patients and among different classifications of brain injury

Type of asynchrony	All patients (*n* = 100)	Types of brain injury			P^*^
		Stroke (*n* = 44)	Post-craniotomy (*n* = 37)	Traumatic brain injury (*n* = 19)	
All types	96 (96%, 92%–100%)	43 (98%, 93%–100%)	35 (95%, 87%–100%)	18 (95%, 84%-100%)	0.670
Ineffective triggering	95 (95%, 91%–99%)	42 (96%, 89%–100%)	35 (95%, 87%–100%)	18 (95%, 84%–100%)	>0.999
Double-triggering	79 (79%, 71%–87%)	33 (75%, 62%–88%)	30 (81%, 68%–94%)	16 (84%, 66%–100%)	0.690
Auto-triggering	6 (6%, 1%–11%)	3 (7%, 0%–15%)	2 (5%, 0%–13%)	1 (5%, 0%–16%)	>0.999
Flow insufficiency	12 (12%, 6%–19%)	7 (16%, 5%–27%)	1 (3%, 0%–8%)	4 (12%, 1%–41%)	0.085
Premature cycling	42 (42%, 32%–52%)	15 (34%, 20%-49%)	16 (43%, 27%-60%)	11 (58%, 33%-82%)	0.221
Delayed cycling	31 (31%, 22%–40%)	13 (30%, 16%–44%)	13 (35%, 19%–51%)	5 (26%, 5%–48%)	0.782
Reverse triggering	5 (5%, 1%–9%)	3 (7%, 0%–15%)	0 (0)	2 (11%, 0%–26%)	0.221

Data are shown as n (%, 95% confidence interval)

^*^Comparison among different classifications of brain injury

### Association of asynchrony with ventilatory mode, respiratory drive and analgesia/sedation

During waveform datasets recording, four ventilatory modes were used, including PSV (65%), PACV (19%), SIMV + PS (10%), and VACV (6%). The proportion of ventilatory mode in each type of asynchrony is shown in (Additional file 1: Figure S1). For 503 datasets with a single asynchrony type, triggering (ineffective, double and auto-triggering) and cycling asynchrony were predominantly detected during PSV (70% to 100%). All single occurred flow insufficiency was detected during VACV, while all single occurred reverse triggering was detected during assist/control modes (PACV 53% and VACV 47%). The proportion of modes in datasets with multiple asynchrony types (n = 403) was 62%, 17%, 16% and 5% during PSV, PACV, SIMV + PS, and VACV, respectively.

**Fig. 4:**
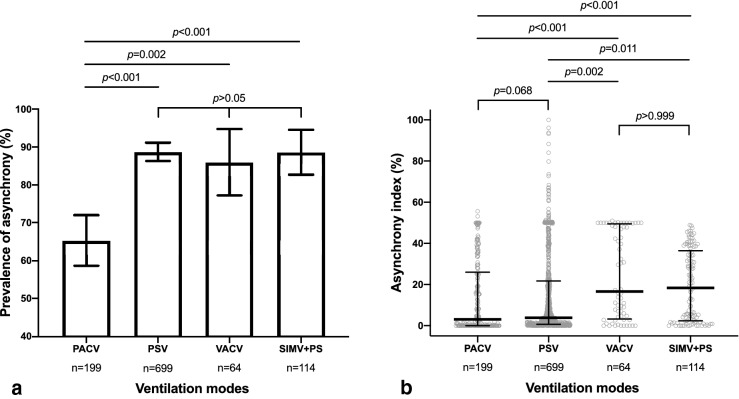
A significant difference in the prevalence of asynchrony was found among different ventilatory modes (*p *< 0.001, panel **a**). Percentage and 95% confidence interval are shown. The prevalence of asynchrony during pressure assist/control ventilation (PACV) was significantly lower than those during pressure support ventilation (PSV), volume assist/control ventilation (VACV), and pressure-preset synchronized intermittent mandatory ventilation plus pressure support (SIMV + PS). No significant difference was found among the PSV, VACV, and SIMV + PS (panel **b**). Individual data, median, and interquartile ranges are shown. A significant difference in asynchrony index was also found among different ventilatory modes (*p *< 0.001, panel **b**). Asynchrony indexes during PACV and PSV were significantly lower than those during VACV and SIMV + PS

Patient–ventilator asynchrony detected in datasets with PACV (65%) was significantly less than those with PSV (89%), VACV (86%) and SIMV + PS (89%) (*p* values ranged from < 0.001 to 0.003), but no significant difference was found among the latter three modes (*p* values ranged from 0.538 to > 0.999) (Fig. 4a).

A significant difference in the asynchrony index was also found among the four ventilatory modes (*p *< 0.001), with significantly lower indexes during PACV and PSV compared to those during VACV and SIMV + PS (*p* values ranged from < 0.001 to 0.011) (Fig. 4b).

**Fig. 5:**
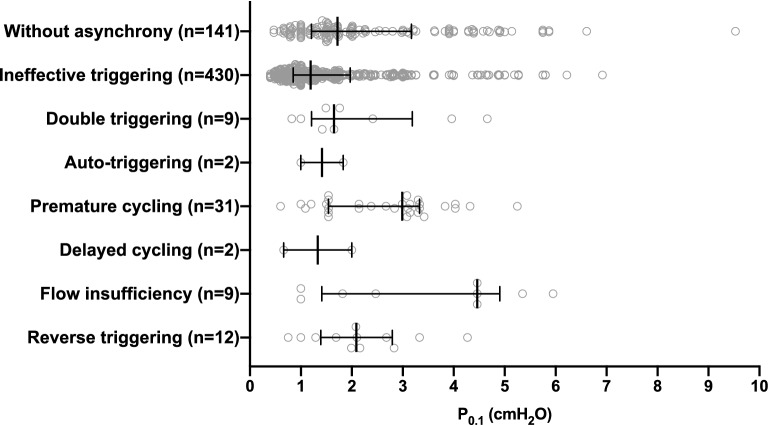
The airway occlusion pressure (P_0.1_) with different types of asynchrony. Individual data, median, and interquartile ranges are shown. Compared to the datasets without asynchrony, P_0.1_ was significantly lower in datasets with ineffective triggering (*p *< 0.001). Although there was a tendency in elevated P_0.1_ values in datasets with premature cycling and flow insufficiency, no statistical significances were found (*p *> 0.999)

Spontaneous breathing was preserved in 1037 (96%) datasets during recording. P_0.1_ values in datasets with a single type of asynchrony are shown in Fig. 5. Compared to the datasets without asynchrony (n = 141, 1.7 [1.2–3.1] cmH_2_O), P_0.1_ was significantly lower in datasets with ineffective triggering (n = 430, 1.2 [0.9–2.0] cmH_2_O, *p *< 0.001). Although there was a tendency in elevated P_0.1_ values in datasets with premature cycling (n = 31, 3.0 [1.5–3.3] cmH_2_O) and flow insufficiency (n = 9, 4.5 [1.4–4.9] cmH_2_O), no statistical significance was found (*p *> 0.999).

**Fig. 6:**
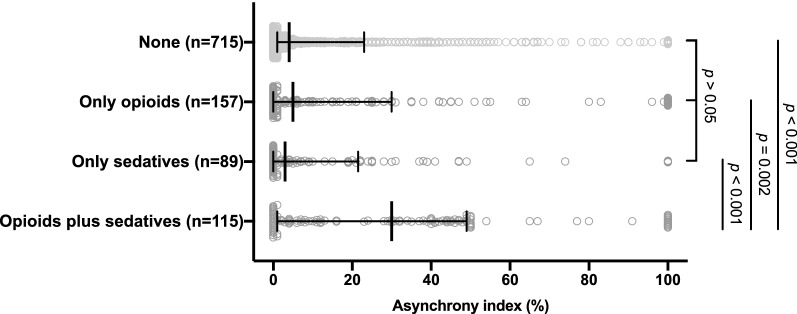
A significant difference in the asynchrony index was found among different analgesia/sedation strategies (*p *< 0.001). Individual data, median, and interquartile ranges are shown. Asynchrony index during combined administration of opioids and sedatives was significantly higher than those during single use of opioids or sedatives, as well as no use of these two types of drugs. No significant difference was found in asynchrony index among the latter three conditions

Asynchrony index in datasets with the combined use of opioids and sedatives was significantly higher than those with single use and no use of these two kinds of drugs (Fig. 6). For analysis of specific drugs, the asynchrony index was significantly higher during the combined use of fentanyl and midazolam, compared to that during single administration of midazolam (*p* < 0.001) or remifentanil (*p* = 0.025) as well as that without opioids and sedatives administration (*p* = 0.016) (Additional file 1: Fig. S2). No significant difference was found in asynchrony index either between datasets with SAS ≤ 2 and ≥ 3 (median [IQR] of 5.2% [0.4%–36.8%] vs. 4.8% [0.7%–26.0%], *p* = 0.827) or between datasets with GCS ≤ 8 and ≥ 9 (median [IQR] of 4.0% [0.4%–31.1%] vs. 5.9% [1.0%–27.0%], *p* = 0.124).

### Associated factors with severe ineffective triggering

In the dataset-based analysis, 275 (26%) datasets were defined as severe ineffective triggering (asynchrony index ≥ 10%), and associated factors included SIMV + PS mode compared to PSV mode, a lower respiratory rate, a higher tidal volume, lower minute ventilation, the use of flow-trigger with a more sensitivity setting, a lower P_0.1_, a higher GCS, and higher arterial partial pressure of carbon dioxide and concentration of bicarbonate (Additional file 1: Table S3).

In datasets collected during pressure-preset ventilatory modes (PACV, PSV and SMIV + PS), set inspiratory pressure was significantly higher in the severe ineffective triggering group than that in the non-severe group (median [IQR] of 10 [8–12] vs. 10 [10–12] cmH_2_O, *p *< 0.001). In datasets undergoing VCAV, tidal volume was likely to preset higher in the severe ineffective triggering group (median [IQR] of 7.3 [6.8–8.0] vs. 7.5 [7.3–8.2] ml/kg IPW), but with no statistical significance (*p *= 0.095).

Severe ineffective triggering was diagnosed in 31 (31%) patients. Only a longer duration of ventilation before the inclusion was found in the severe ineffective triggering group (median [IQR]: 20 [16–23] vs. 19 [15–21] hours, *p *= 0.050). Duration of ventilation after the inclusion and length of stay in the hospital was significantly longer in patients with severe ineffective triggering (Additional file 1: Table S4).

## Discussion

The main findings of the present study are: (1) asynchrony is detected in almost all enrolled brain-injured patients with a breath-based prevalence of 38%; (2) the most prevalent type of asynchrony is ineffective triggering; (3) ventilatory mode, respiratory drive and analgesia/sedation strategy are associated asynchrony; and (4) severe ineffective triggering is related to prolonged duration of mechanical ventilation and length of stay in hospital. To the best of our knowledge, this is the first study to investigate the patient–ventilator asynchrony in mechanically ventilated brain-injured patients comprehensively.

Several parameters have been used to report the prevalence and the severity of asynchrony, including the proportion of asynchronous breaths in all collected breaths [24], patient-based asynchrony index with a single recording dataset [20, 21] or merging of multiple recording datasets [9], and asynchrony index in long-term collected data [25, 26]. The proportion of asynchronous breaths in our brain-injured patients (38% in total inspected breaths of 330,292) was higher than that reported in 27 ICU patients without brain injury (23% in 43,758 breaths) [24]. Because ventilatory modes and settings, as well as the patient’s conditions and pharmacological treatments, change throughout the entire course of mechanical ventilation, asynchronies usually occur in a cluster pattern [27, 28]. Therefore, patient-based prevalence and severity derived from a single short-term dataset may not represent the real-world situation. Although several algorithms have been designed to detect patient–ventilator asynchrony in a big database automatedly, dedicated software is preferred [29]. In the present study, we introduced periodical datasets collection to facilitate the analysis of the relationship of asynchrony prevalence and severity with ventilatory and respiratory factors.

In line with previous reports, ineffective triggering was also found as the most prevalent type of asynchrony in our group of brain-injured patients. The ineffective triggering is relatively easy to detect by analyzing flow and airway pressure tracings [1, 2]. However, given its high prevalence, sensitive instruments, such as monitor or software with continuous detection function, are warranted for screening the mechanically ventilated patients [28, 29]. On the other hand, it is challenging to confirm reverse triggering, flow insufficiency and cycling abnormalities by only observing the flow and airway pressure waveforms. The accurate detection of these asynchronous types is mostly dependent on the use of special monitoring, such as esophageal pressure [11, 12]. Our results showed that all these types of asynchrony occurred in our group of brain-injured patients, suggesting the selected use of additional monitoring in high-risk populations.

Complex interactions among ventilatory modes, settings, and respiratory drive are related to patient–ventilator asynchrony. Our data demonstrated a lower prevalence of asynchrony during PACV and a lower severity during PACV and PSV, compared with those during VACV and SIMV + PS (Fig. 4). It may be reasonable to conduct PACV in the acute phase and change to PSV to protect spontaneous breathing in patients with acute brain injury. Asynchrony types are newly classified as the difference between the patient’s respiratory drive and the ventilator’s supply [1, 2]. In the present study, we determined P_0.1_ and found a lower respiratory drive in the presentation of ineffective triggering and a higher tendency in premature cycling and flow insufficiency (Fig. 5). In accordance with previous reports [23], a higher inspiratory pressure was set in the dataset with ineffective triggering. It was also found that reducing tidal volume during PSV by lowering pressure support level markedly decreased the severity of ineffective triggering [30]. These findings suggest stratified ventilation strategies for patients with different respiratory drives.

Analgesia and sedation may also a potential factor associated with asynchrony. Previous studies showed that different analgesics and sedatives promoted different asynchronous events [31, 32]. A recent study focusing on the influence of analgesia found that the use of opioids alone was associated with less patient–ventilator asynchrony [25]. In the present study, we found that asynchrony was more severe during the combined use of opioids and sedatives, with the most notable for the combination of fentanyl and midazolam (Fig. 6 and Additional file 1: Figure S2). Early studies also found that deeper sedation level was a predictor of ineffective triggering [33] and increasing sedation depth was insufficient on double-triggering compared with adjusting the ventilatory settings [34]. In brain-injured patients, we did not find the association of overall asynchrony severity with either consciousness or sedation depth. Because indications of sedation and analgesia treatment also include the control of intracranial pressure and lower cerebral metabolism in patients with brain injury [14], the association of sedation with asynchrony warrants further investigation.

In the present study in acute brain-injured patients, an association of severe ineffective triggering with a prolonged duration of mechanical ventilation and hospital length of stay was found. This is comparable to the early studies in general ICU patients with a relatively short time interval of data collection [23, 35]. Although the design of the study cannot assess the cause–effect relationship, the potential association between asynchrony and outcome suggests further in-depth researches.

There are limitations to the study. First, this was a single-center study. Because asynchrony can be influenced by treatment modality, our findings may not be generalized to other units. Second, we only enroll brain-injured patients with primary diagnoses of stroke, post-craniotomy for brain tumors and traumatic brain injury. The results may not represent the situations in other types of injury. Nevertheless, the three injury types we included compose the majority of brain injury admitted to the ICU. Third, asynchrony was visually detected during offline analysis. However, we used esophageal pressure monitoring to facilitate the diagnosis of asynchrony. Additionally, the offline analysis was performed independently by two investigators, and group discussion was employed as the two readings were discrepant. This guarantees the accuracy of the diagnosis.

## Conclusions

Patient–ventilator asynchrony is common in acute brain-injured patients undergoing mechanical ventilation. The most prevalent type of asynchrony is ineffective triggering. Although the classification of brain injury is not a factor related to asynchrony, ventilatory modes, respiratory drive and analgesia/sedation strategies are associated with patient–ventilator asynchrony, which suggests the need to adjust treatment for this specific population.

## Supplementary information

**Additional file 1:** Definition of asynchrony, and tables and figures.

## Data Availability

The datasets used and/or analyzed during the current study are available from the corresponding author on reasonable request.

## Abbreviations

APACHE: Acute Physiology and Chronic Health Evaluation
CI: Confidence interval
FiO_2_: Inspired oxygen fraction
GCS: Glasgow Coma Scale
GOS: Glasgow Outcome Scale
ICU: Intensive care unit
P_0.1_: Airway occlusion pressure
PACV: Pressure assist/control ventilation
PaO_2_: Arterial partial pressure of oxygen
PEEP: End-expiratory positive pressure
PSV: Pressure support ventilation
SAS: Sedation–Agitation Scale
SIMV + PS: Pressure-preset synchronized intermittent mandatory ventilation plus pressure support
SOFA: Sequential organ failure assessment
VACV: Volume assist/control ventilation
