# Supplementary material for: Patient–ventilator asynchrony in acute brain-injured patients: a prospective observational study
Source: Ann Intensive Care. 2020 Oct 19;10:144. doi: 10.1186/s13613-020-00763-8 (PMC7570406; doi:10.1186/s13613-020-00763-8)
Supplement: Supplementary file 1 — Additional file 1: Definition of asynchrony, and tables and figures. [file 13613_2020_763_MOESM1_ESM.pdf]

Patient-ventilator asynchrony in acute brain-injured patients: a prospective observational study

Xu-Ying Luo, Xuan He, Yi-Min Zhou, Yu-Mei Wang, Jing-Ran Chen, Guang-Qiang Chen, Hong-Liang Li, Yan-Lin Yang, Linlin Zhang, Jian-Xin Zhou

Additional file 1

Contents

- ☐ Definition of patient-ventilator asynchrony and example waveforms
- ☐ **Table S1** Inter-observer reliability for the detection of asynchrony
- ☐ **Table S2** Asynchrony index in enrolled patients (n=100)
- ☐ **Figure S1** Proportion of ventilatory modes in different types of asynchrony
- ☐ **Figure S2** Asynchrony index with administration of different opioids and sedatives
- ☐ **Table S3** Factors associated with severe ineffective triggering on the basis of dataset analysis
- ☐ **Table S4** Factors associated with severe ineffective triggering on the basis of patient analysis

Definition of patient-ventilator asynchrony and example waveforms

Seven asynchrony patterns were determined through a priori definition, including ineffective triggering, double triggering, auto-triggering, flow insufficiency, premature cycling, delayed cycling, and reverse triggering [S1-S5].

References

S1. Pham T, Telias I, Piraino T, Yoshida T, Brochard LJ: Asynchrony Consequences and Management. Crit Care Clin 2018; 34:325-341

S2. Esperanza JA, Sarlabous L, de Haro C, Magrans R, Lopez-Aguilar J, Blanch L: Monitoring Asynchrony During Invasive Mechanical Ventilation. Respir Care 2020; 65:847-869

S3. Georgopoulos D, Prinianakis G, Kondili E: Bedside waveforms interpretation as a tool to identify patient-ventilator asynchronies. Intensive Care Med 2006; 32: 34-47

S4. Murias G, Lucangelo U, Blanch L: Patient-ventilator asynchrony. Curr Opin Crit Care 2016; 22: 53-59

S5. Antonogiannaki EM, Georgopoulos D, Akoumianaki E: Patient-Ventilator Dyssynchrony. Korean J Crit Care Med 2017; 32: 307-322

S6. de Haro C, Lopez-Aguilar J, Magrans R, Montanya J, Fernandez-Gonzalo S, Turon M, Goma G, Chacon E, Albaiceta GM, Fernandez R, Subira C, Lucangelo U, Murias G, Rue M, Kacmarek RM, Blanch L, Asynchronies in the Intensive Care Unit G: Double Cycling During Mechanical Ventilation: Frequency, Mechanisms, and Physiologic Implications. Crit Care Med 2018; 46: 1385-1392

S7. Nilsestuen JO, Hargett KD: Using ventilator graphics to identify patient-ventilator asynchrony. Respir Care 2005; 50: 202-234

S8. de Wit M: Monitoring of patient-ventilator interaction at the bedside. Respir Care 2011; 56: 61-72

S9. Gilstrap D, MacIntyre N: Patient-ventilator interactions. Implications for clinical management. Am J Respir Crit Care Med 2013; 188:1058-1068

Abbreviations

Paw: airway pressure; Pes: esophageal pressure

1. Ineffective triggering [S1-S3, S5]

Ineffective triggering is defined as that a negative deflection in esophageal pressure is presented but ventilator does not deliver inspiration. Ineffective triggering can occur during either expiratory or inspiratory phase. The following figure shows the waveforms during pressure assist/control ventilation. The patient has three attempts that do not trigger the ventilator. Two arrows A indicate ineffective triggering during expiratory phase. Arrow B indicate ineffective triggering during inspiratory phase. The red line indicates the onset of inspiratory effort.

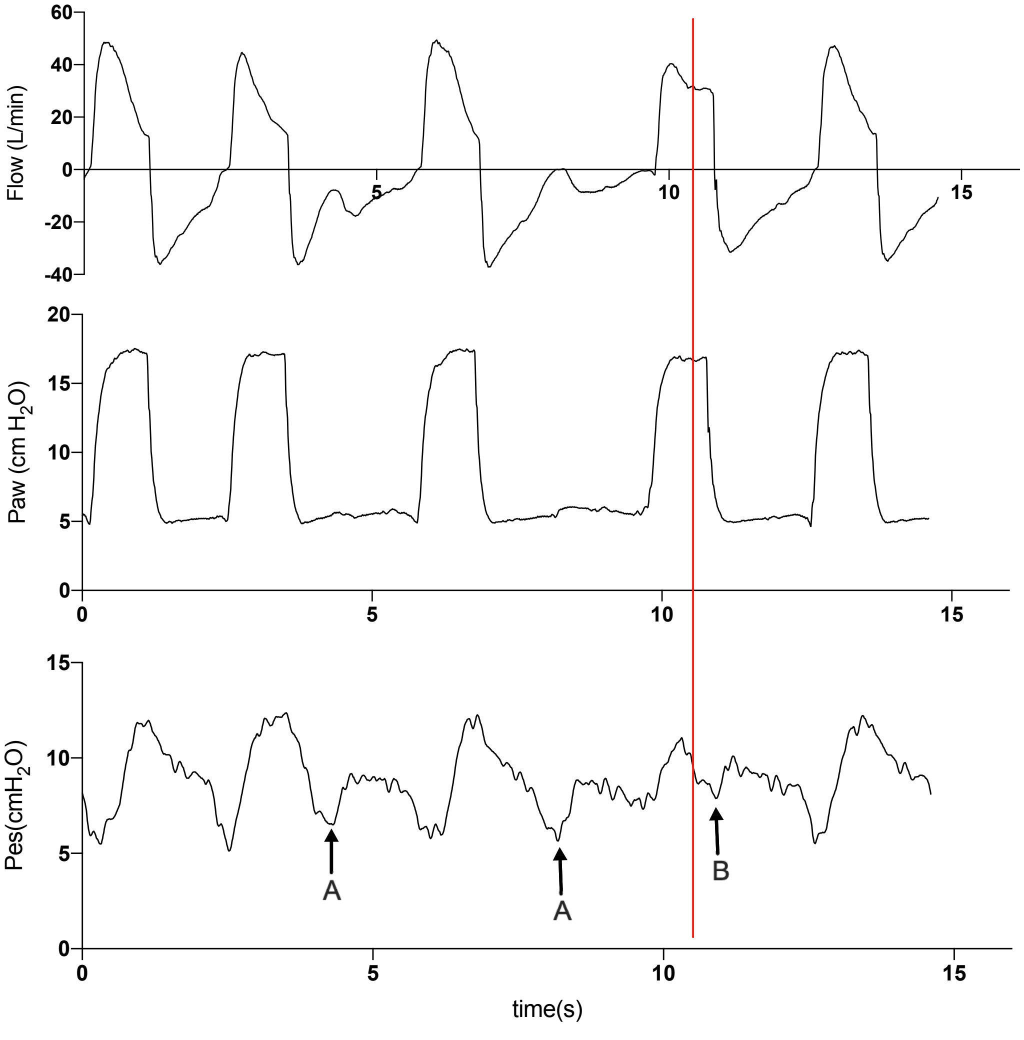

2. Double triggering [S3, S6]

Double triggering is defined as that two complete inspirations separated by a very short expiratory time, which is usually resulted from the patient’s inspiratory time longer than the ventilator’s delivered inspiratory time. The persistence of the patient’s inspiratory effort triggers the ventilator a second time without allowing the completion of expiration. The following figure shows the waveforms during volume assist/control ventilation. Red line indicates the cycling of the ventilator, but the patient’s inspiratory effort continues.

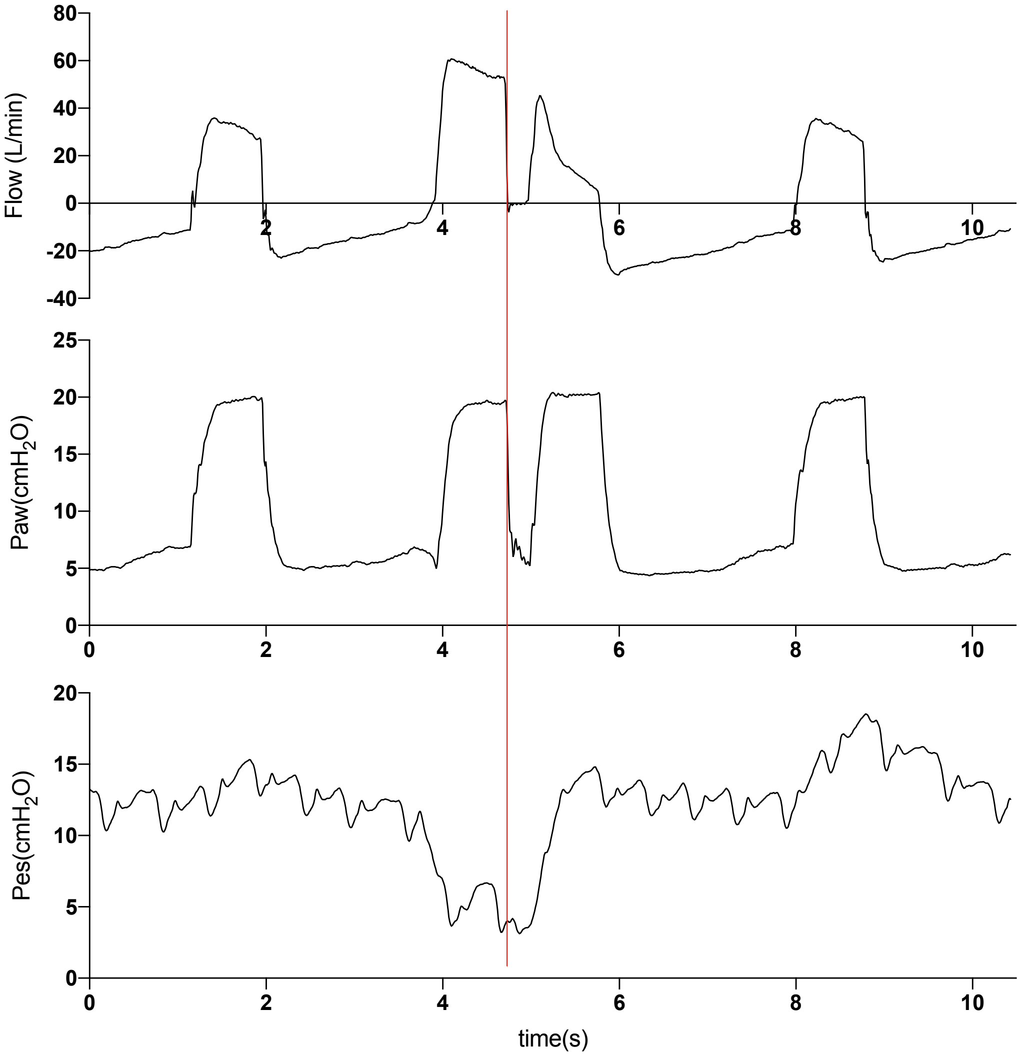

3. Auto-triggering [S3, S5]

Auto-triggering is defined as the ventilator being triggered in the absence of patient's effort. The following figure shows the waveforms in patient with deep coma during pressure assist/control ventilation. The respiratory rate was set as 10 breaths/min. The monitored respiratory rate was 15 breaths/min. No negative deflection can be found at the beginning of inspiration. End-expiratory occlusion did not induce the patient's spontaneous inspiration. This case of auto-triggering was resulted from leak after checking the ventilator circuit.

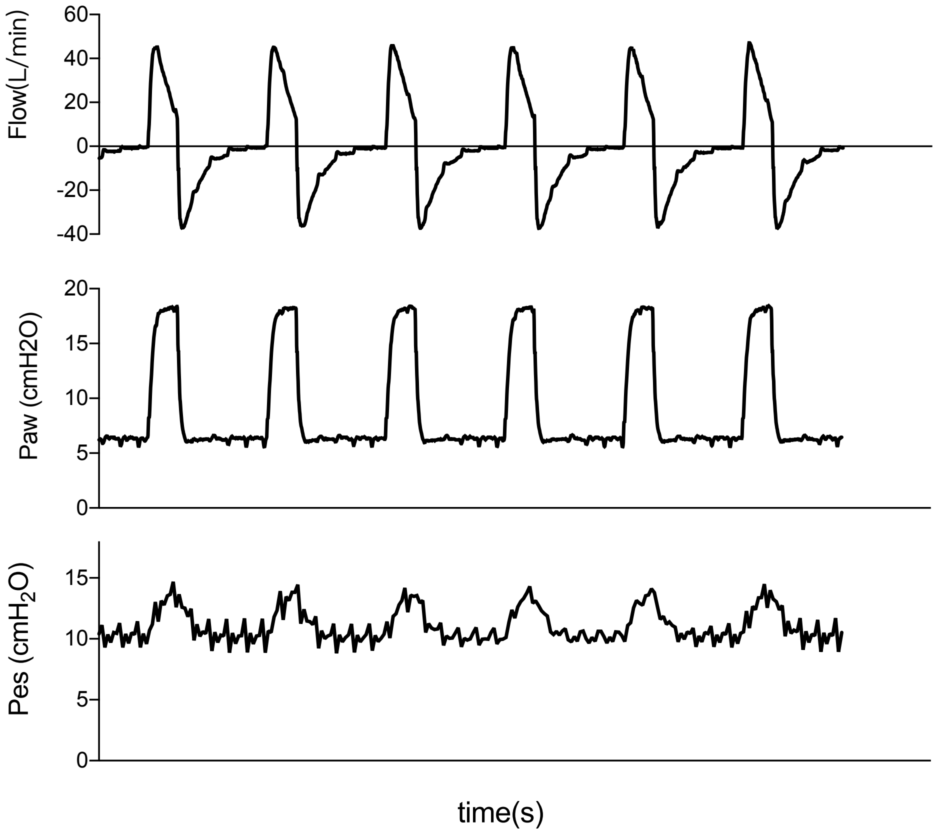

4. Flow insufficiency [S4, S7-S9]

Flow insufficiency is defined as that inspiratory flow delivered by the ventilator does not fully meet the patient's inspiratory demand. The following figure shows the waveforms during volume assist-control ventilation. It can be found a concave deflection in the airway pressure tracing during the inspiratory phase (red arrows). Blue arrows indicate relatively large negative deflection in the esophageal pressure tracing representing the patient's effort.

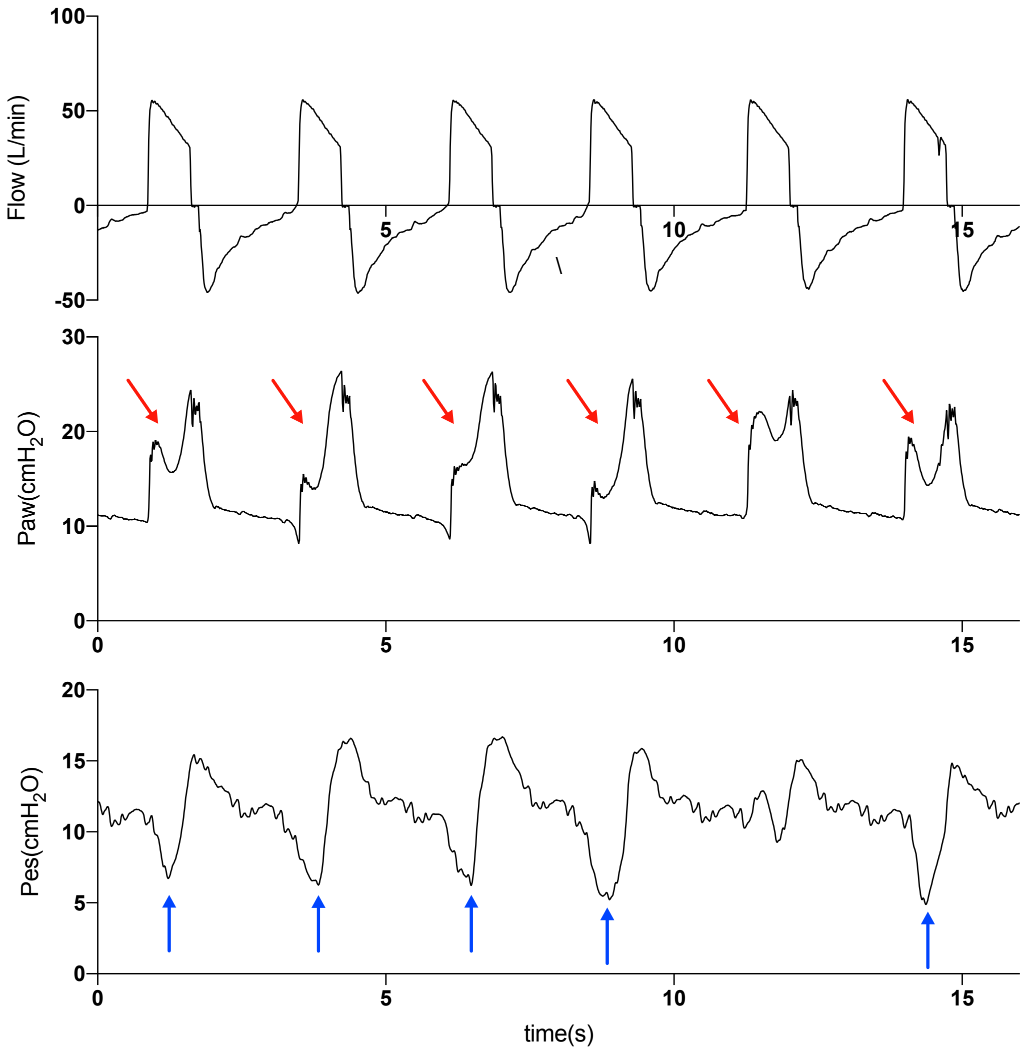

5. Premature cycling [S1, S3]

Premature cycling is defined as that the patient's inspiratory effort continues during the ventilator cycles from inspiration to expiration. The following figure shows the waveforms during pressure support ventilation. After the ventilator's cycling (red line), the patient's inspiratory effort continues indicated by the negative deflection of the esophageal pressure tracing (arrows).

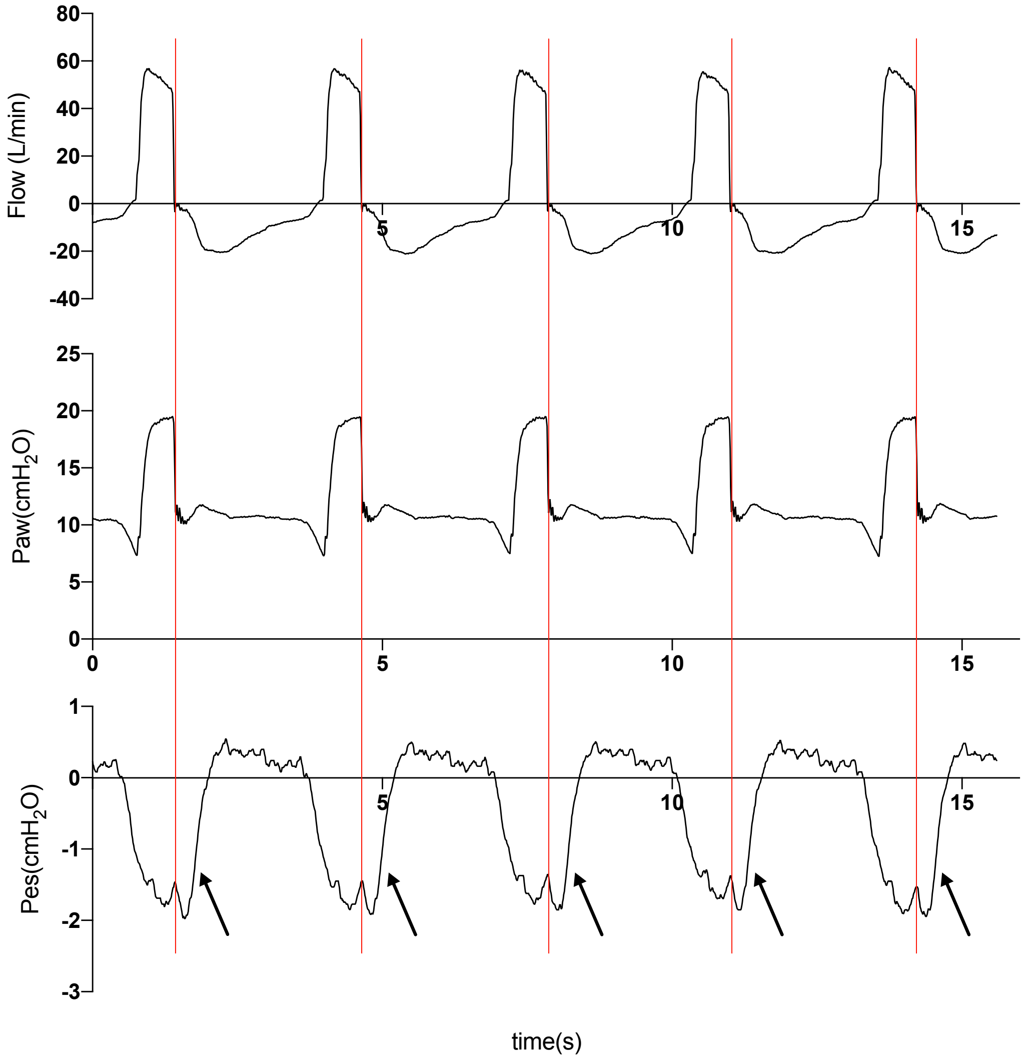

6. Delayed cycling [S1, S3-S5]

Delayed cycling is defined as that the ventilator delivery of inspiration continues after the patient's inspiratory effort has ceased. The following figure shows the waveforms during pressure support ventilation. The red line indicates the ventilator cycling. The blue line indicates the termination of patient's inspiratory effort which can be measured as the esophageal pressure increasing to 70% of the maximal negative deflection. An overshoot in airway pressure tracing can be found at the end of inspiration (arrows).

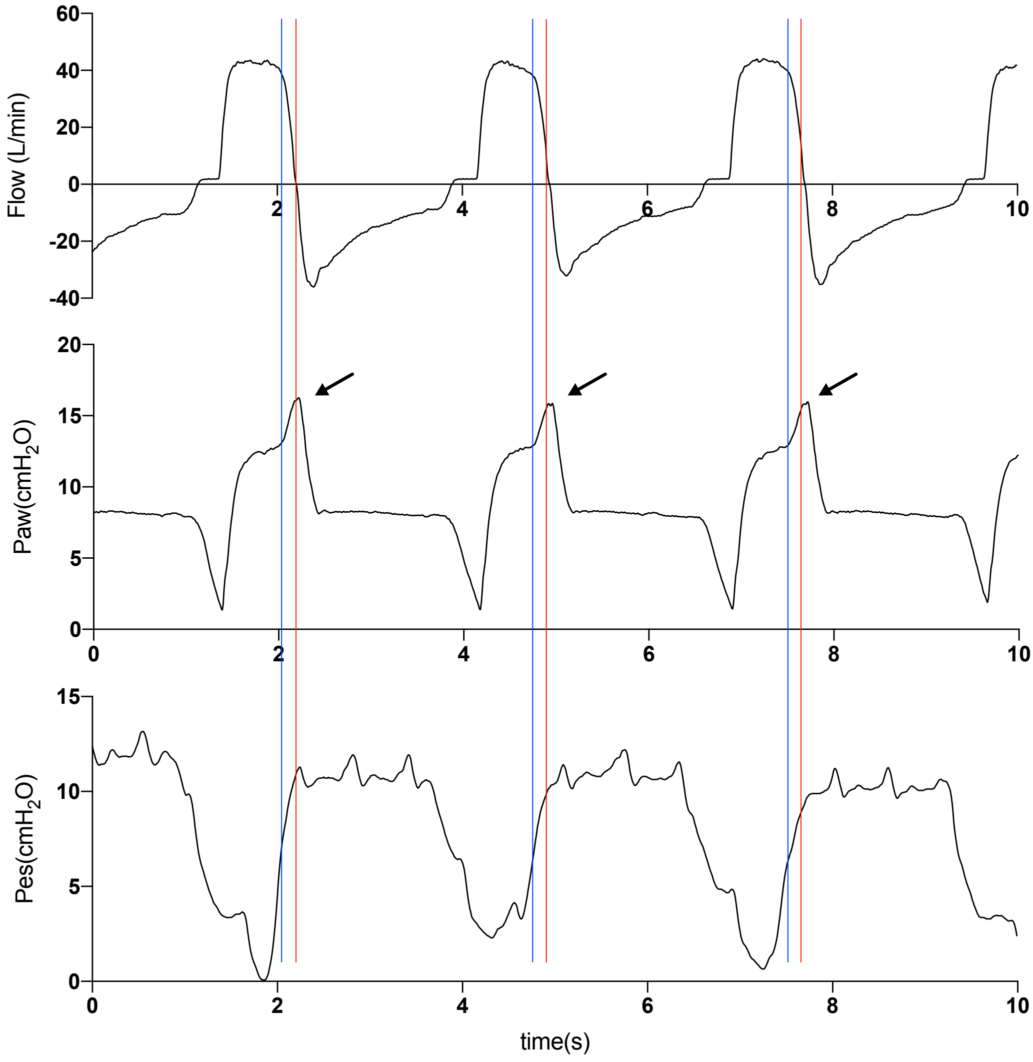

7. Reverse triggering [S1, S4]

Reverse triggering is defined as the ventilator triggered patient's muscular effort, which occurs during controlled ventilation. The following figure shows the waveforms during volume assist/control ventilation. Arrows show the patient's inspiratory efforts triggered by the ventilator in a repetitive and consistent manner. Blue arrows show reverse triggering without breath stacking; and the red arrow shows a relative stronger inspiratory effort triggered by the ventilator, which causes a second breath delivering (breath stacking).

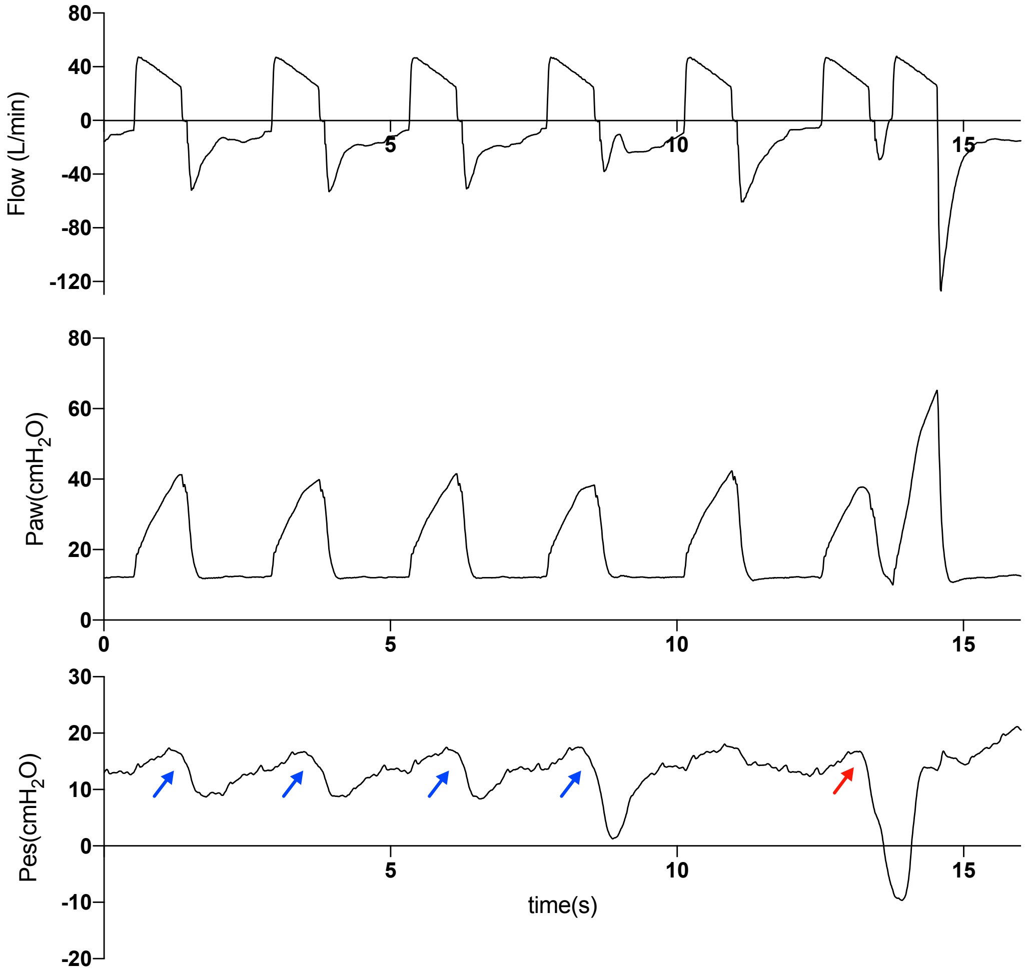

Table S1 Inter-observer reliability for the detection of asynchrony

Type of asynchrony	Number of breaths detected by observer A	Number of breaths detected by observer B	Number of breaths finally confirmed	Weighted kappa (95% CI)
Ineffective triggering	34941	34134	35003	0.986 (0.985–0.987)
Double triggering	1801	1697	1851	0.956 (0.949–0.963)
Auto-triggering	1547	1641	1672	0.991 (0.987–0.994)
Flow insufficiency	3821	4581	4277	0.965 (0.961–0.969)
Premature cycling	20363	16877	19282	0.930 (0.927–0.933)
Delayed cycling	4208	3450	3858	0.944 (0.938–0.949
Reverse triggering	4014	4390	4213	0.979 (0.976–0.982)

95% CI confidence interval

Table S2 Asynchrony index in enrolled patients (n=100)

Type of asynchrony	Asynchrony index %, median (IQR)
All types	12.4 (4.3–26.4)
Ineffective triggering	4.3 (1.9–11.8)
Double triggering	0.2 (0.1–0.5)
Auto-triggering	7.7 (2.7–12.6)
Flow insufficiency	3.8 (1.1–11.8)
Premature cycling	1.8 (0.2–11.5)
Delayed cycling	0.6 (0.2–4.8)
Reverse triggering	15.0 (7.2–24.3)

IQR interquartile range

Figure S1 Proportion of ventilatory modes in different types of asynchrony

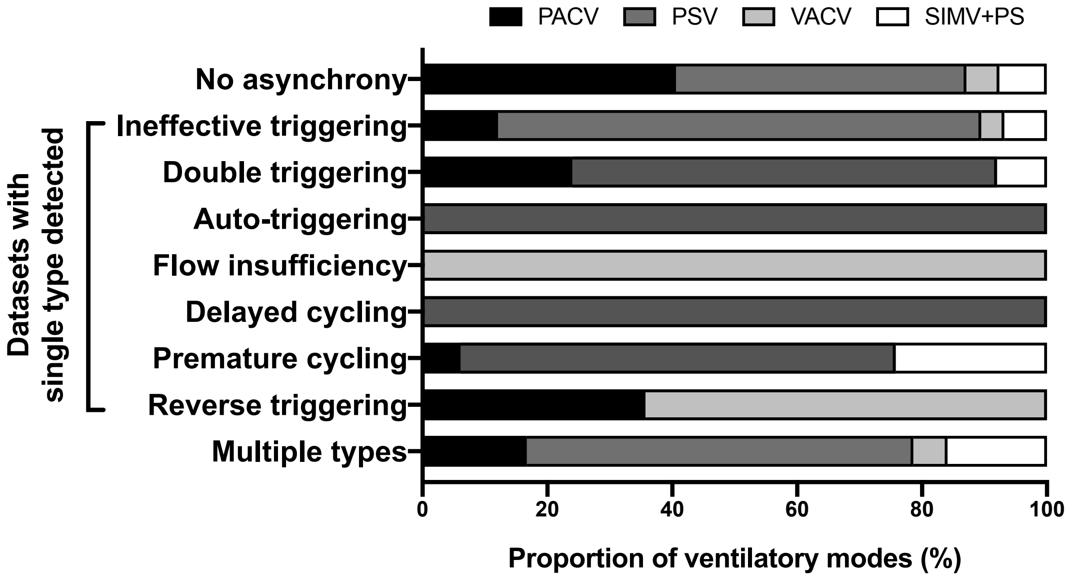

Abbreviations

PACV pressure assist/control ventilation, *PSV* pressure support ventilation, *VACV* volume assist/control ventilation, *SIMV+PS* pressure-preset synchronized intermittent mandatory ventilation plus pressure support

Figure S2 Asynchrony index with administration of different opioids and sedatives

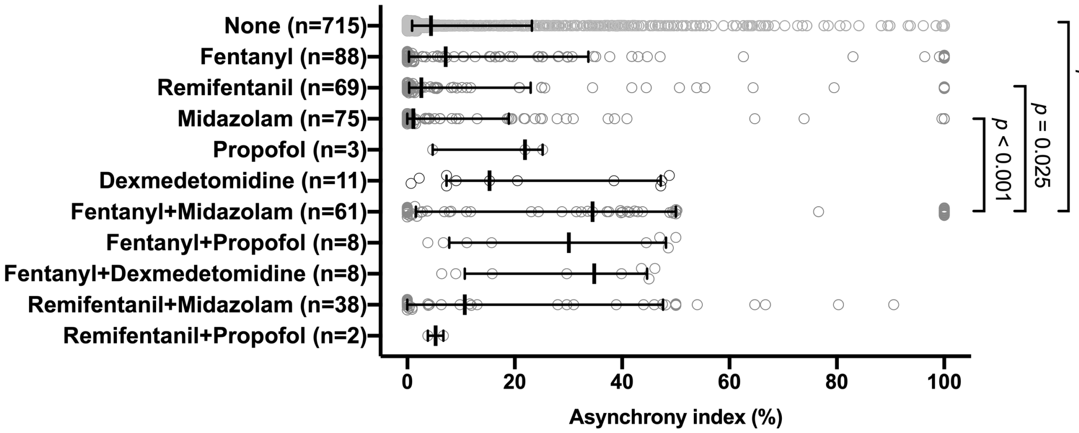

Data are shown as individual values, median and interquartile range.

A significant difference was found in asynchrony index in datasets with administration of different opioids and sedatives ($p < 0.001$). Asynchrony index was significantly higher during combined use of fentanyl and midazolam, compared to that during single administration of midazolam ($p < 0.001$) or remifentanil ($p = 0.025$) as well as that without opioids and sedatives administration ($p = 0.016$). There was no significant difference among other pairwise comparisons ($p > 0.05$).

Table S3 Factors associated with severe ineffective triggering on the basis of dataset analysis

	AI ≥10% (n=275)	AI <10% (n=801)	*p*
Ventilatory mode, *n* (%)			0.027
PACV	56 (20.4)	143 (17.9)	
PSV	163 (59.3)	536 (66.9)	
VACV	15 (5.5)	49 (6.1)	
SIMV+PS	41 (14.9)	73 (9.1)	
Ventilatory parameters			
Respiratory rate, breaths/min	15 (13–19)	18 (15–21)	<0.001
Tidal volume, ml/kg IPW	8.1 (7.2–9.8)	8.0 (7.0–9.2)	0.024
Minute ventilation, L/min	7.7 (6.2–9.9)	8.9 (7.7–10.9)	<0.001
PEEP, cmH_2O	6 (5–8)	5 (5–8)	0.297
Type of trigger, *n* (%)			0.001
Flow	230 (83.6)	588 (73.4)	
Pressure	45 (16.4)	213 (26.6)	
Flow trigger sensitivity, L/min	2 (2–3)	3 (2–3)	<0.001
Pressure trigger sensitivity, cmH_2O	1.0 (1.0–1.5)	1.0 (1.0–1.5)	<0.001
$P_{0.1}$, cmH_2O	1.2 (0.9–2.0) (n=274)	1.6 (1.0–2.7) (n=763)	<0.001
GCS	10 (6–11)	9 (6–11)	0.001
SAS	3 (2–4)	3 (2–4)	0.101
Use of sedatives and/or opioids, *n* (%)			0.063
None	197 (71.6)	518 (72.4)	
Only opioids	27 (9.8)	130 (16.2)	
Opioids plus sedatives	29 (10.5)	86 (10.7%)	
Only sedatives	22 (8.0)	67 (8.4)	
Blood gas analysis	(n=100)	(n=313)	
pH	7.46 (7.42–7.49)	7.47 (7.43–7.50)	0.121
$PaCO_2$, mmHg	42 (36–46)	37 (34–41)	<0.001
HCO^{3-}, mmol/L	29 (26–31.3)	27.3 (25.5–29.8)	0.007
PaO_2/FiO_2, mmHg	269 (198–362)	246 (195–336)	0.173

Categorical variables are shown as number (percentage); continuous variables are shown as median (interquartile range).

FiO_2 inspired oxygen fraction, *GCS* Glasgow Coma Scale, *$P_{0.1}$* airway occlusion pressure, *PACV* pressure assist/control ventilation, *PaO_2* arterial partial pressure of oxygen, *PEEP* end-expiratory positive pressure, *PSV* pressure support ventilation, *SAS* Sedation-Agitation Scale, *SIMV+PS* pressure-preset synchronized intermittent mandatory ventilation plus pressure support, *VACV* volume assist/control ventilation

Table S4 Factors associated with severe ineffective triggering on the basis of patient analysis

	AI≥10% (n=31)	AI<10% (n=69)	*p*
Male sex, *n* (%)	19 (61.3)	48 (69.6)	0.416
Age, yr	55 (39-67)	52 (39-63)	0.288
Body mass index, kg/m^2	24.2 (21.6-25.8)	24.2 (21.2-26.6)	0.500
Classification of brain injury, *n* (%)			0.829
Stroke	13 (41.9)	31 (44.9)	
Post-craniotomy for brain tumors	11 (35.5)	26 (37.7)	
Traumatic brain injury	7 (22.6)	12 (17.4)	
APACHE II at the ICU admission	19 (14-22)	18 (14-21)	0.820
SOFA at the inclusion	5 (4-7)	6 (5-8)	0.327
GCS at the inclusion	10 (6-11)	8 (5-11)	0.290
Duration of mechanical ventilation before the inclusion, h	20 (16-23)	19 (15-21)	0.050
Acute hypoxemic respiratory failure, *n* (%)	22 (71)	54 (78.3)	0.430
PaO_2/FiO_2 ratio at inclusion, mmHg	250 (173-393)	223 (176-295)	0.226
Duration of mechanical ventilation after the inclusion, days	10 (6-21)	6 (5-12)	0.020
ICU length of stay after the inclusion, day	19 (10-41)	16 (10-22)	0.158
Length of stay in hospital after the inclusion, day	39 (27-48)	27 (23-37)	0.006
GOS at the end of follow-up	3 (2-3)	3 (2-3)	0.280
All-cause mortality, *n* (%) (in-hospital mortality)	4 (12.9)	12 (17.4)	0.770

Categorical variables are shown as number (percentage); continuous variables are shown as median (interquartile range). *APACHE* Acute Physiology and Chronic Health Evaluation, FiO_2 inspired oxygen fraction, *GCS* Glasgow Coma Scale, *GOS* Glasgow Outcome Scale, *ICU* intensive care unit, PaO_2 arterial partial pressure of oxygen, *SOFA* Sequential Organ Failure Assessment
